# Editorial Department

**Published:** 1859-04

**Authors:** Gideon B. Smith


BALTIMORE, March 25, 1859.

*Messrs. Editors:*

Having now had considerably over a year's experience with artificial teeth mounted on the cheoplastic principle, I can speak with more assurance than I could in my former letter. The piece I wear is a complete set for the under jaw, and I am now able to say that it is every thing I could wish. During the last twelve years I have had several sets of teeth mounted after the other most approved methods, but none have given me the satisfaction I now enjoy with the cheoplastic set. I would not exchange it indeed, for the most perfect one on gold if it were offered me for the one I now wear, which appears to be so durable that I have no doubt it will last me during my life. The metal has no taste and affords no lodgment for particles of food or other substances, and the adaptation is exact; so perfect is it in this latter respect that I prefer this method for greater adaptability, to all others. I am constantly in the habit of recommending the process to my patients and friends. Very respectfully,

GIDEON B. SMITH, M. D.

